# Research on the Process and Influencing Factors of Online Diabetes Information Users’ Avoidance Behavior: A Qualitative Study

**DOI:** 10.3390/bs13030267

**Published:** 2023-03-17

**Authors:** Caiqiang Guo, Li Si, Yifan Sun

**Affiliations:** School of Information Management, Wuhan University, Wuhan 430072, China

**Keywords:** online diabetes information avoidance, information encountering, qualitative study, model, process, factors

## Abstract

Users’ avoidance behavior of health information has received growing attention recently, but research into users’ avoidance behavior of diabetes information remains limited. This paper aims to reveal the process and the factors of avoiding online diabetes information. The interview, conducted with the critical incident technique, and the diary methods were used to collect 40 true incidents of online diabetes information avoidance from 17 participants. Based on the thematic analysis method and grounded theory, the data were analyzed to identify the key phases of the avoidance process and obtain the factors influencing the occurrence of avoidance behavior. The results showed that the macro-process of online diabetes information avoidance comprised three phases: pre-encountering, encountering, and avoiding after encountering. First, browsing, searching, or social interaction provide the context for encountering; second, the encountering occurrence consists of three steps—noticing the stimuli, reacting to stimuli, and examining the content; and third, to avoid the online diabetes information encountered, users will adopt avoidance strategies, such as avoiding information sources, controlling attention, delaying access, forgetting information, and denying information, which is manifested as general avoidance and strong avoidance, and has positive, negative, or no effect on users. The 14 influencing factors of avoidance behavior obtained were divided into four clusters. User-related factors include demographic characteristics, health-behavior perception, perceived threat, perceived control, and information sufficiency; information-related factors include information quality, information overload, and information dissemination; environment-related factors include context type, behavior place, time pressure, and social factors, and emotion-related factors include the pre-encountering and post-encountering emotional states. These findings can guide the intervention of information avoidance behavior.

## 1. Introduction

According to the 10th edition of the International Diabetes Federation diabetes atlas, diabetes has become one of the fastest-growing global health concerns in the twenty-first century. Currently, 537 million adults worldwide have diabetes, with a prevalence rate of 10.5% [1]. Diabetes complications affect multiple organs and lead to high disability and mortality rates. In 2021, about 6.7 million adults died of diabetes or its complications, accounting for 12.2% of the global death toll [1]. China had about 140 million diabetics in 2021, ranking first in the world. Diabetes prevalence among adults increased from 4.2% in 2002 to 11.2% in 2017 [2,3]. Although the awareness, treatment, and control rate of diabetes improved in recent years, they remained low [2]. It is urgent to improve the ability of people with pre-diabetes to obtain appropriate and timely care, effectively preventing or delaying complications, avoiding premature death, and improving their quality of life. Access to online health information is an essential way for information consumers to improve their quality of life and meet their information needs.

Active access to health information helps individuals understand their health status and medical diagnosis, and maintain their health [4]. However, a large body of research has found that people do not always actively access health information and sometimes deliberately avoid it to maintain or increase their level of uncertainty [5,6]. “Health information avoidance” is a prevalent issue. According to research on the information behavior of people with life-threatening diseases, such as breast cancer, prostate cancer, AIDS, etc., some individuals avoid learning about their health status [7,8,9]. Although some studies have confirmed that reasonable avoidance of health information can temporarily alleviate negative emotions such as anxiety and fear [10], in the long run, it affects people’s perception of their health status and may lead to missed opportunities to detect diseases early and improve lifestyle habits [11].

Online health information avoidance is the behavior presented by individuals of avoiding or delaying access to available online health information [11]. Some researchers put information avoidance behavior in the context of information seeking [12,13], and less in that of information encountering. The relevant research on the behavior of information encountering only considered the accidental discovery and active acquisition of unexpected information and ignored the instinct of human beings to generate avoidance motivation for external stimuli, thus paying less attention to the avoidance behavior after information encountering [14]. Online health information encountering is useful or interesting health information that users encountered while searching and browsing, which can help users solve past, present, or future health problems related to themselves or others [14]. Moreover, it plays an important role in disease prevention, diagnosis and treatment, can optimize health decisions for users, improve or change health behaviors, and better play the value of health information. Avoiding such information means that individuals would lose out on early detection of diseases and the correction of unhealthy lifestyles, which is not conducive to the flow of information and the realization of information value. Although there has also been research on whether individuals were willing to accept the detection of type 2 diabetes and diabetics’ avoidance behavior of health information [15,16,17], the avoidance of diabetes information received less attention. Therefore, it is necessary to explore users’ avoidance behavior of online diabetes information.

This study puts information avoidance in the context of information encountering, aiming to solve the following questions:

**RQ1.**  What are the major phases that constitute an online diabetes information avoidance process?

**RQ2.**  What happens during each constituting phase in the online diabetes information avoidance process?

**RQ3.**  What are the major factors that influence the occurrence of online diabetes information avoidance?

**RQ4.**  How does each influencing factor act on the occurrence of online diabetes information avoidance?

## 2. Literature Review

Health information avoidance research mainly focused on the content and influencing factors of information avoidance, and a few studies have explored the avoidance strategies and results.

### 2.1. Research on Influencing Factors of Health Information Avoidance

Relevant studies mainly focused on the avoidance of disease health information, including cancer [9,10], diabetes [15,16,17], and daily health information, such as skin damage [18] and physical exercise [19]. The respondents included patients [17], college students [15], females [9,20], pregnant women [21], elderly people [22], rural residents [23], and other different groups.

Researchers explored the factors that influenced users’ avoidance behavior of health information in three aspects: user-related factors, information-related factors, and context-related factors. (1) User-related factors: female, elderly, low-income, low-education, and low information literacy users tended to avoid health information related to subjects such as cancer, physical exercise, and epidemic [19,24,25]. Another study found that younger users were more likely to avoid information [26]. Negative emotions, such as fear, anxiety, worry, etc., led to users’ avoidance behavior of cancer information [10,22,27]. However, the direction of the relationship between worry and cancer information avoidance was inconsistent in China and the U.S. [28]. Perceived risk promoted users’ avoidance behavior of health information [6,23]. When users believed that the disease was controllable, they would avoid health information less [9]. Compared with people with more coping resources, people with fewer were more likely to avoid information [29]. Cognitive dissonance increased the willingness to avoid information [30], but self-efficacy decreased individuals’ willingness [31,32]. Individual self-affirmation helped reduce the avoidance of risk information, but increased users’ avoidance when information may force people to carry out unwanted behaviors and when information was related to the risk of an untreatable disease [33]. (2) Information-related factors: researchers found that information overload aggravated users’ cognitive burden, triggered negative emotions such as anxiety and fatigue, and led to avoidance behavior [13,25,34,35]. Source credibility, information utility and characteristics had an impact on users’ avoidance behavior [21,23,36]. In addition, the expression of health information titles affected users’ avoidance behavior. Compared with loss frame titles, gain frame titles attracted more and longer attention and more clicks [20]. (3) Context-related factors: health information avoidance was situational, relatively common, not necessarily unhealthy, and may be used to achieve multiple communication goals [37]. People avoided health information mainly to maintain hope or deniability, resist overexposure, accept limits of action, manage flawed information, maintain boundaries, and continue with life/activities [37]. Social norms, behavior changes, task driving, and society influenced users’ avoidance behavior [12,21,36].

### 2.2. Research on Avoidance Strategies

To achieve the purpose of avoiding information, people often adopted certain avoidance strategies, such as avoiding information sources, controlling attention, delaying access, forgetting information, and denying information. (1) Avoiding information sources. In real life, people often avoided unwanted information by avoiding specific information sources, including newspapers, books, websites, social networking platforms, television programs, and other people or institutions that may provide information [38]. (2) Controlling attention. Even if people had obtained information from information sources, they were often unable to focus on information. When information conflicted with their cognition and caused discomfort, people avoided the information by diverting or distracting their attention [39]. People may choose to pay attention to positive information and remain indifferent to adverse or threatening information, even though such information may be more useful [40]. (3) Delaying access. When people were unable to process information immediately due to cognitive or emotional factors but were aware that it had potential value, they tended to delay accessing it to gain enough time to adjust their cognition or emotional state [41]. Then, they tried to understand the information that had not been obtained after adjusting their state [42]. (4) Forgetting information. Even though people obtained the information, they still deliberately and selectively did not recall negative information [40]. (5) Denying information. When people tried to forget information and failed, they could have a biased understanding or interpretation of the information, thus rejecting and denying the original meaning of information transmission [37,40].

### 2.3. Research on Avoidance Results

Less attention has been paid to the effect of information avoidance on respondents, and the conclusions vary. Some pointed out that information avoidance brought on negative effects, while others believed that it brought on positive effects. (1) Negative effect. The most direct result of information avoidance makes people miss the opportunity to eliminate uncertainty and optimize decision making [11]. Individuals with health risks often avoided medical examinations, which made people miss opportunities for prevention, early detection of diseases, making better decisions, obtaining better treatment, and improving lifestyle habits [43]. (2) Positive effect. When people could not or believed they could not change their environment and status quo, information avoidance could help them increase cognitive uncertainty, alleviate cognition disorders and negative emotions such as fear and anxiety [35], or maintain their original state and firm up their existing decisions or plans [37]. Maintaining or increasing uncertainty could make patients optimistic and increase comfort, which is conducive to treatment and enables patients to temporarily live a “happy life” [6,40].

At present, the related researches mainly focus on the factors influencing the occurrence of health information avoidance behavior, devoting less attention to avoidance strategies and results, and there is a lack of research on “how avoidance behavior occurs”. Researchers pay less attention to diabetes information. Information encountering often occurs in the online environment. This paper tries to put avoidance behavior in the context of information encountering, to explore the users’ avoidance behavior of online diabetes information, and reveal the process and influencing factors of avoidance behavior, to comprehensively grasp how and why the users’ avoidance behavior occurs.

## 3. Method

### 3.1. Research Method Selection

Because information avoidance was not easy to observe directly, the interview method was mainly used to collect qualitative data in previous studies, and the diary method was rarely used [11]. The diary method is a self-report method of obtaining and recording the subject’s immediate feelings. Compared with the interview, the diary method can help researchers collect dynamic and real-time data from respondents, effectively shorten the time interval of recall, reduce the risk of retrospective bias, and ensure the authenticity of research results [44,45]. The interview method is also advantageous in obtaining details of user behavior and mental activity, and it can be used alone or as a complement to the diary method. Therefore, the interview method, with the critical incident technique, and the diary method were adopted to collect qualitative data on users’ avoidance behavior when they encountered online diabetes information.

### 3.2. Respondents Selection and Recruitment

In the previous research on the avoidance behavior of disease information, researchers mainly focused on patients. Obtaining disease information facilitates not only timely diagnosis, treatment, and daily health care for patients, but also health management and disease prevention for non-patients. The patient’s family members understand the relevant knowledge, which can supervise the patient’s treatment and daily health care to help control the patient’s condition. Therefore, this study investigated diabetic patients, family members of diabetic patients, and general users to obtain a comprehensive understanding of users’ avoidance behavior. The authors recruited respondents on different platforms such as the Post bar of Wuhan University, WeChat moments, Diabetes forum, QQ group of diabetes, etc.

### 3.3. Data Collection

The data were collected from 14 January to 31 March 2022. First, the authors told the participants what “online diabetes information they encountered” was, what “the avoidance of online diabetes information they encountered” was, and several forms of avoidance of online diabetes information. After that, the authors asked them to keep a diary for two weeks, providing them with diary points and diary examples. The diary points included the time, place, scene, emotional state, activities, platform, whether there was a clear demand before information encountering, the way of information encountering, the topic and characteristics of the information encountered, the reaction to the information encountered, the reason for avoidance, the strategy, result, and intensity of avoidance behavior, behavior after avoidance, etc. Participants needed to record the avoidance behavior in a diary when it occurred. A minimum of 150 words were required to be recorded. Participants were informed that a minimum number of diary entries was not required and not related to the final reward. After they completed their diaries, the authors conducted supplementary interviews with them to address what was unclear in the diaries and interviewed them again based on the interview outline which was the same as the diary points to obtain their experience of avoidance behavior. Affected by the COVID-19 epidemic, the interviews were completed through WeChat voice. The authors told participants who only participated in the interview that we would record the interview content for the convenience of subsequent data collation, desensitize them in the subsequent processing, and record them after obtaining consent. The authors adjusted the content of the interview according to the actual situation to collect relevant data on the avoidance behavior of the respondents. The length of each interview was 15–30 minutes.

Seventeen respondents (encoded as P1–P17) participated in the diary and interview data collection methods, including eleven females and six males. Because there were diabetics around or in their families, the respondents were often concerned or willing to learn about diabetes-related information. Among them, there were two diabetic patients, eleven family members of diabetic patients, and four general users, aged between 19 and 32, with college, undergraduate, master, and doctorate education backgrounds. Their majors included library science, information science, information management and information system, management science and engineering, accounting, chemistry, communication engineering, law, clinical medicine, etc.

### 3.4. Data Pre-Processing

Seventeen respondents contributed forty-seven incidents (encoded as I1–I47), forty-two of which come from diaries and five come from interviews. Since most respondents were first exposed to the concepts of “information encountering” and “information avoiding”, the phenomena of “false information encountering” and “false information avoiding” was inevitably included. To ensure the accuracy and effectiveness of this study, the authors screened according to the following criteria: (1) whether the participants encountered online diabetes information under unexpected situations, (2) whether the online diabetes information they encountered was useful or interesting for the participants, (3) whether the participants avoided the information they encountered under unexpected situations, and (4) excluding the records of avoidance made by the participants due to advertising information. Unfortunately, seven of the incidents were detected to be false, as attributable to the following reasons:

① It does not belong to information encountering: I38 and I46. Users discovered online diabetes information during their active search and had expectations. The user (P13) actively searched for “diet and resting to prevent diabetes” on Weibo (I38), and the user (P16) searched for “will low blood glucose turn into high blood glucose” on Baidu (I46). The search content had a high correlation with diabetes, and users had expectations for the search results of the two incidents.

② It belongs to information encountering: I20, I41, I42, I43, and I44. Users (P7 and P15) encountered interesting or useful information in unexpected circumstances and checked the content. These instances belonged to information encountering, but they did not trigger information avoiding.

### 3.5. Data Analysis Method

The qualitative thematic analysis method included identifying, analyzing, and reporting patterns or topics in data [46]. Inductive thematic analysis was based on empirical data and data-driven methods. Grounded theory was employed to find new phenomena that had not been mentioned in existing research [46]. The grounded theory allowed researchers to theoretically explain the general characteristics of the topic through associative patterns, while the explanation was based on the empirical observation of the data through categorical coding.

Therefore, this study used inductive thematic analysis to analyze and extract the topics involved in the process of avoidance behavior. The authors followed the basic analysis steps of thematic analysis, that is, getting familiar with data, generating initial coding, formal coding, and summarizing topics [46]. The authors conducted the thematic analysis as follows: (1) the researchers read all the descriptions carefully and took notes for each meaningful unit; (2) initial codes were created based on these notes; (3) the relationships between the initial codes were analyzed, and similar ones were combined to generate formal codes; (4) formal codes were refined or incorporated to form sub-themes and main themes. The authors used grounded theory to analyze and refine the influencing factors of avoidance behavior and followed the three-level coding process of open coding, spindle coding, and selective coding. The goal of open coding is to extract initial concepts from the original material; the goal of spindle coding is to develop main categories based on open coding by refining and merging the initial categories; and the goal of selective coding is to further develop core categories based on the main categories.

## 4. Results

### 4.1. Phase of the Avoidance Behavior Process

The authors analyzed the remaining 40 true incidents based on thematic analysis to identify the key phases of the avoidance behavior process. Table 1 provides three examples of the coding process. Simply speaking, a note was a keyword or phrase captured in original descriptions, an initial code was a higher-level abstraction of the phenomenon reflected in a note, and a formal code unified several similar initial codes. The authors annotated meaningful sentence fragments, marking 387 nodes in total, generating 136 initial codes, such as QQ Zone, Weibo, and WeChat official account. After comparing and combining the initial codes, 93 official codes such as QQ, Weibo, and WeChat were finally formed.

Then, the authors analyzed the relationship between formal codes, classified and merged them according to their similarity in meaning, and formed 43 sub-themes, including social media, search engines, video platforms, etc. Finally, according to the meaning expressed by the sub-themes and the correlation between them, the authors obtained 15 main themes, such as the online environment, pre-encountering emotional state, and foreground activities. The sub-themes were second-level themes. According to the sequence of occurrence, they could be divided into three stages: pre-encountering, encountering, and avoiding after encountering, as shown in Table 2.

The authors found two characteristics of avoidance behavior: dynamic variability of avoidance intensity and avoidance behavior. Dynamic variability of avoidance intensity included from general to strong avoidance, from strong to general avoidance, and avoidance behavior depending on the specific situation. Dynamic variability of avoidance behavior included from avoidance to non-avoidance, from non-avoidance to avoidance, unchanged, and avoidance intensity depending on the specific situation.

#### 4.1.1. Pre-Encountering

The participants’ everyday life (N = 29), study (N = 7), and work (N = 1) provided the context for their online diabetes information encountering. Three incidents (e.g., I19, I29, I30) were missing activity scenes. Life-related scenes, such as shopping (e.g., I5, I7), socializing (e.g., I6), searching for health knowledge (e.g., I11, I33), or just killing time (e.g., I1, I3, I4), accounted for the absolute majority of incidents. In contrast, relaxing during doing homework (e.g., I26), reading literature (e.g., I31, I35), writing an opening report (e.g., I36), or solving problems users encountered during the study (e.g., I37) were typical study-related scenes. In work-related scenes, users mainly killed time during breaks (e.g., I15).

The foreground activities before the information encountering mainly included browsing (N = 33), searching (N = 5), and social interaction (N = 2). The term “browsing” was used here in a broad sense. It may be that users opened mobile devices to view the latest information, or used social media (N = 24), video platforms (N = 10), search engines (N = 3), etc. to consume various information. Browsing aimlessly, without specific goals, was especially helpful for information encountering (e.g., I1, I2, I3). In contrast, prior activities were identified in five of these incidents. To solve problems in everyday life and studying (e.g., I11, I33, I35, I36, I37), users searched and then encountered the information, even when the user’s explicit target was covered by an entirely different implicit target. Social interaction was found in everyday life-related scenes such as chatting (e.g., I6) and playing games (e.g., I10). Instant messaging services such as WeChat had promoted the information exchange of network users when establishing or maintaining online social relations.

The users’ emotional state of pre-encountering influenced their reaction and attitude to health information, which could be classified as positive or negative. Positive emotional states were mainly manifested in leisure, happiness, relaxation, etc., while negative emotional states were mainly manifested in sadness, grief, pain, etc. Users were mostly in positive emotional states (N = 32) and less in negative emotional states (N = 7) in pre-encountering. It was easier to avoid the online diabetes information encountered in a negative emotional state.

#### 4.1.2. Encountering

Users’ avoidance behavior began when they noticed stimulus elements in the information that they encountered, which caused users to deviate from the original exploratory behavior and make cognitive and behavioral responses according to their cognition and needs [47].

The information content of pre-encountering and encountering mainly had two types: ① When users browsed or searched irrelevant content on health, such as WeChat Moments, news, foods, painting, etc., users encountered diabetes information (e.g., I1, I3, I7). ② Users encountered diabetes information when browsing or searching for general health information, such as fat reduction and renal failure (e.g., I11, I35).

Stimulus noticed. The topic and presentation of the online diabetes information encountered attracted users’ attention. The topics of the online diabetes information encountered included diagnosis and examination, treatment, daily health care, complications, social life, prevention, scientific research, etc. The topics of complications and scientific research were more likely to be avoided by users than other topics. Most users felt that the content related to complications was too uncomfortable. Users avoided scientific research due to their poor readability (e.g., I11, I15, I23). Users read or watched text, pictures, videos, and other forms of content.

Reaction to stimulus. After noticing the online diabetes information they encountered, users matched their cognition and needs with their knowledge and the needs of themselves or others and responded behaviorally. Users immediately avoided or further accessed the online diabetes information encountered. (1) Users immediately avoided online diabetes information (N = 13), including avoiding information sources and delaying access. When the online diabetes information encountered caused users discomfort, such as panic, irritability, rejection, etc., users would immediately avoid information sources (e.g., I4, I13, I34). When the user was inconveniently obtaining the online diabetes information encountered due to time or place constraints, he would delay access (e.g., I36, I39). (2) Users further accessed the online diabetes information encountered (N = 27). When the user thought that the online diabetes information he encountered was useful or interesting, he would access the online diabetes information further. Users would examine the specific content when they found the title of the online diabetes information attractive and useful to them or others, guiding patients in their medication and satisfying their knowledge needs (e.g., I8, I12). When users found the information interesting or novel, it would arouse their curiosity and drive them to click on the content to examine it (e.g., I15, I23).

Content examined. Even if users examined the online diabetes information they encountered, it did not mean they would accept and use it. The user’s surroundings, user information access preferences, and content affected the user’s reaction and emotional state after examining the online diabetes information he encountered, which in turn affected subsequent behavior. If the readability of the online diabetes information they encountered was poor, it would reduce users’ interest in reading, resulting in users’ distraction or withdrawal from the current read (e.g., I21). Practicality also had an impact on users’ judgment about the value of the information encountered (e.g., I10). When information caused users cognitive conflict, users would choose to avoid the current information (e.g., I31). After examining the online diabetes information encountered, it gave rise to users’ positive or negative emotional states. Positive emotional states motivated users to use the information further (e.g., I24, I27, I28), while negative emotion tended to lead to avoidance behaviors (e.g., I11, I35).

#### 4.1.3. Avoiding after Encountering

After information encountering, users adopted some strategies to avoid the information encountered due to cognitive imbalance or emotional discomfort to reduce the impact of the information encountered on them [11].

Avoidance strategies and their manifestations. Confronted with the online diabetes information encountered, users adopted some strategies, such as avoiding information sources (N = 17), controlling attention (N = 13), delaying access (N = 5), forgetting information (N = 7), and denying information (N = 1). It should be noted that the user may adopt multiple avoidance strategies in an incident. For example, the user first avoided information sources and then forgot information (e.g., I45). Even if users adopted the same avoidance strategy, its manifestation could also be different. (1) The manifestations of avoiding information sources included directly swiping these away, staying away from information sources (N = 10), quitting reading information (N = 2), withdrawing applications (N = 3), turning off mobile phones (N = 1), etc. (e.g., I1, I4, I9). (2) The manifestations of controlling attention included quickly browsing the information content by distracting one’s attention (N = 8) (e.g., I4, I6), examining some content that conformed to the current stage (N = 3) (e.g., I11), or only being distracted by other content (N = 2) (e.g., I35), even if other contents were also useful. (3) The manifestations of delaying access. Users delay access to information when they encountered it at a time where there was time pressure or inconvenient access to the information. Users found the information useful for themselves and (or) patients, but they had difficulty remembering or implementing it (e.g., I27, I28, I39). They would record, collect, save, share, etc., for subsequent access (e.g., I27, I28). (4) The manifestations of forgetting information. A: The information was useful, but it was not useful at the current stage. Users selectively forgot or directly ignored the information encountered (e.g., I10). B: The information was useful, but it caused users discomfort or disturbed users’ current life state. Users chose to forget the information encountered to maintain the current state (e.g., I35). C: The information was useful, but the information content or suggestions was not easy to practice. Users chose to ignore the information (e.g., I15). D: The information was useful, but users felt that the complications of diabetes were not easy to prevent. Users did not want to think of it (e.g., I29). E: The information was useful, but it was different from the user’s actual situation, so the user no longer remembers it (e.g., I40). (5) The manifestations of denying information. When the information content or suggestion obtained was inconsistent with the user’s inherent ideas or habits, the user would deny them to maintain his current behavior (e.g., I31).

Avoidance intensity. It included general avoidance and strong avoidance. In most cases, users generally avoided it based on the information and its content (e.g., I33). Compared with daily health care, prevention, and treatment of diabetes, complications and professional academic research were more likely to cause strong avoidance by users (e.g., I35). When users were in group activities, in public places, or depressed, they were more likely to avoid the health information encountered (P15).

The effect of avoidance behavior. Its positive effects were mainly manifested in reducing worry and discomfort, improving the efficiency of information acquisition, and reducing cognitive burden (P2). The negative effect was mainly manifested in reducing the acquisition of health knowledge, which was not conducive to disease prevention (P7). For family members of diabetic patients and general users, who thought that they could not implement the content or suggestions of online diabetes information by themselves, or they were in good health, with low information demand, avoiding the online diabetes information encountered had little or no effect on them (P9).

The emotional state and behavior after avoiding. Avoiding information might maintain or reverse users’ emotional state, strengthen or weaken their initial emotions, showing positive or negative emotional states, which further affects their subsequent behavior. They performed the following behaviors: ① Returning to the initial activity before information encountering (N = 12); ② Ending all activities (N = 7); ③ Using the information encountered (N = 3); ④ Further exploring (N = 3).

In addition, users’ avoidance behavior had two characteristics, including the dynamic variability of avoidance behavior and avoidance intensity. Vertically, user behavior was dynamic. It changed from avoidance to non-avoidance, and from non-avoidance to avoidance. Once the avoidance preference has been formed, it will not change in a short time. (1) From avoidance to non-avoidance, users (P4 and P10) pointed out that they had never learned about information related to diabetes (i.e., blood glucose) in the past and believed that only people with diabetes needed to be concerned about some information, so they had been avoiding it. After they inadvertently learned about their utility, they no longer avoided such information. Therefore, it is feasible and necessary to promote and disseminate diabetes knowledge among general users and family members of diabetic patients. In addition, users decide whether to avoid online diabetes information depending on the stage they are in. (2) From non-avoidance to avoidance, this occurred mainly for specific information sources and information content. When users (P12) knew that certain information sources were unreliable or had low information value, they would avoid information on that source. In addition, users’ avoidance behavior varied with their health status, cognitive level, knowledge needs, information content, and the environment when faced with the same topic or content from an information source, depending on the specific situation. The dynamic variability of users’ avoidance behaviors suggests that interventions can be made to improve users’ utilization of information resources and facilitate information flow. However, some users’ avoidance behaviors are unvarying. For topic-specific content or information from a specific information source, users always avoid it. Users’ willingness to avoid has a dynamic change from general avoidance to strong avoidance and from strong avoidance to general avoidance, depending on the context (P15).

### 4.2. Factors Influencing the Occurrence of Avoidance Behavior

Based on the idea of grounded theory, the authors extracted 246 original sentences and corresponding initial concepts from the initial material, generating 86 initial concepts, such as practical, academic, and useful. After comparing and combining the initial concepts, 37 categories were finally formed, such as practicability, readability, and usefulness. Table 3 provides three examples of the coding process. Analyzing and integrating those categories, 15 main categories were formed, such as information quality, information overload, and information dissemination. Finally, according to the meaning of the main categories and their interrelationships, they were summarized into five core categories: information-related factors, user-related factors, environment-related factors, emotion-related factors, and information avoidance behavior, as shown in Table 4.

#### 4.2.1. Information-Related Factors

The authors extracted three information-related factors from the data, including information quality (n = 10), information dissemination (n = 9), and information overload (n = 2). When users perceived that the information quality was not high or not as good as expected, they would avoid the online diabetes information encountered. The information was of poor practicality, low usefulness, poor readability, and unreliable content, consistent with the previous research results [26,41].

*This article contained a lot of arguments in Chinese and English. I learned that sugar was closely related to many diseases, such as diabetes, but this article was too long and had too many confusing arguments (P7).*

*It was very professional. Many academic concepts were very boring for ordinary people to read. I pass it quickly (P12).*

Information dissemination, including information source reliability, information title description, information topic, and information presentation, was related to the realization of information value. Users generally did not directly avoid trusted information sources and information published by certified and authoritative publishers.

*I thought what he said was very authoritative (P4)*.

Users tended to read novel, professional, clear, and rigorous titles and avoided frightening and lengthy titles.

*I usually don’t read those unprofessional titles (P2).*

Whether the information topic and the title could attract users’ attention was directly related to whether the information could be further utilized. From the analysis results, users tended to avoid information about diabetes complications, severe or painful experiences caused by diabetes, and scientific research progress.

*I would always avoid diabetes complications (P1).*

*I wouldn’t look at information about severe experiences caused by diabetes (P2).*

Information in graphic form was easier to be noticed for users.

*If it had pictures, I would read more (* *P4).*

*I thought the text looked intuitive and fast (P10).*

Information overload caused by excessive information feeds and high similarity gave rise to users’ cognitive overload, triggering negative emotions, which led to avoidance. It was consistent with the previous research conclusions [13,25,34].

*I didn’t turn off the notification of the applications such as Quark and Baidu, but they always sent me such notifications. I didn’t have time to look at these notifications... I turned them off without looking at them (P6).*

*I would avoid it due to a large amount of similar information (P2).*

#### 4.2.2. User-Related Factors

The authors extracted five user-related factors from the data, including information sufficiency (n = 5), health-behavior perception (n = 4), demographic characteristics (n = 3), perceived threat (n = 2), and perceived control (n = 1). Information sufficiency was determined by the gap between the information that an individual had (i.e., knowledge reserve) and the amount of information required (i.e., information demand). When the gap was small, the user was prone to avoid it.

*Then I didn’t know much about insulin yet. I want to find out which is better to take medicine or insulin (P6).*

Health-behavior perception was the user’s subjective judgment of the difficulty and utility of health behavior, which affected the user’s willingness to avoid it. If the content or suggestions of the online diabetes information they encountered were easy to implement and had good utility, users would use the information. If it was effective but difficult to implement, users would choose to forget the information.

*But in my current state of life, it was hard to do what it required, so I didn’t think about it anymore (P3).*

*I knew about blood glucose regulation and how it affected diabetes, fat, and the whole body. Then I learned that blood glucose was still important. Later, when I came across information about blood glucose, I would read it too (P4).*

Demographic characteristics affected the users’ perception of health information when they encountered online diabetes information. When the user was in poor health, the user’s needs would increase, which reduced the possibility of avoidance. In addition, personal or family history of disease influenced users’ judgment of online diabetes information.

*Recently, I had backache, decreased immunity, and other conditions. In addition, it was said that diabetes was hereditary. More and more news says that diabetes was at a younger age...Therefore, I was more interested in seeing this information about the blood glucose control experience (P10).*

Perceived threat, including perceived severity and perceived susceptibility, influenced users’ attitudes toward online diabetes information. When the user perceived diabetes susceptibility, the user was more willing to obtain daily health care and prevention information and reduced the possibility of direct avoidance (P6). When users perceived diabetes to be severe, being confronted with the online diabetes information encountered, especially diabetes complications or other serious consequences, increased users’ worry and fear, which promoted the occurrence of avoidance behavior (P2).

Perceived control refers to the user’s perceived control over the information and the consequences of his actions. When the patient perceived that his condition was stable, he was more confident, which would reduce the likelihood of avoiding information about diabetes (P5).

#### 4.2.3. Environment-Related Factors

The authors extracted four environment-related factors from the data, including context type (n = 16), behavior place (n = 2), time pressure (n = 7), and social factors (n = 1).

Context type mainly refers to foreground activities, including browsing, searching, social interaction, studying, working, and everyday life. Compared with browsing, the user was more likely to avoid the online diabetes information encountered while searching and during a social interaction. Users tended to directly seek the target content in the search scene, which reduced the possibility of obtaining the online diabetes information encountered and increased the user’s willingness to avoid it. The online diabetes information encountered by the user interrupted his mental state in the social interaction scenario and thus led to avoidance.

*I didn’t know how to distribute the questionnaire. I began to search the variable scale with the mobile phone. As soon as I opened the homepage, I was pushed a bunch of articles on diabetes treatment and prevention. I should have read such preventive articles before, but now I was too busy to finish my task. Therefore I quickly crossed them away (P13).*

However, most users browsed aimlessly or randomly. When the online diabetes information encountered was useful or interesting, users would further obtain it. With plenty of time in the everyday life scene, users did not directly avoid the online diabetes information encountered, but it was easy to occur in the study and work scene.

*After browsing the Weibo homepage, I was pushed a blog post. Then, I opened the full text and had a look (P2).*

Behavior place. When users were in public places, their behavior was restricted by their surroundings, thus reducing the probability of obtaining information about online diabetes they encountered and increasing their willingness to avoid it.

*When I was relaxing in the office [not alone]... I was interested in the information, but I didn’t bring headphones at that moment. I wanted to collect it and then check it with headphones when I at the dormitory (P13).*

*I would like to see some information that would help me understand the disease, but I didn’t want to see this when there were people around me... If there were a lot of people, I would directly delete it, regardless of whether its content was useful to me or not (P15).*

Time pressure refers to the lack of free time when users encounter online diabetes information, which refers not only to the feeling of not having enough time but also to the emotional experience of being rushed and overwhelmed [47]. When users are busy or in situations of time constraints, they avoid the online diabetes information they encounter.

*Deliberate avoidance, just when you are busy (P5).*

Social factors in this paper referred mainly to subjective norms, that is, the pressure from others or society that users felt while deciding whether or not to examine the online diabetes information they encountered. The impact of subjective norms on avoidance behavior was more pronounced in patients.

*The user mentioned that she didn’t want others to know that she was concerned about online diabetes information and would directly avoid online diabetes information she encountered when in a public place, regardless of whether the information was useful (P15).*

#### 4.2.4. Emotion-Related Factors

The authors extracted two emotion-related factors from the data, including the emotional state of pre-encountering and post-encountering. Positive emotional states promoted user approach behavior, and negative emotional states led to users’ avoidance behavior.

**Figure 1:**
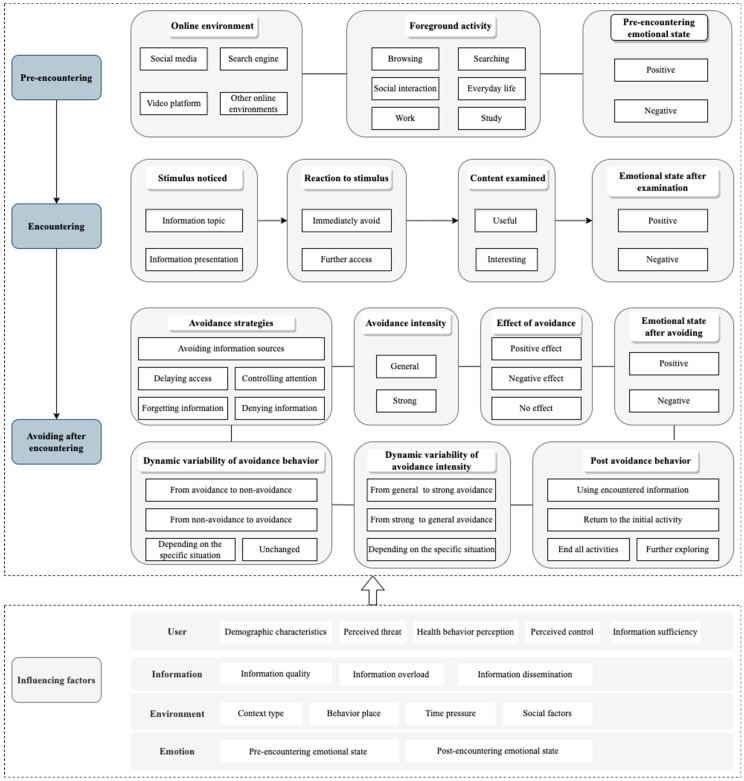
An integrated model of online diabetes information avoiding.

Based on the results of the above analysis, a model of online diabetes information avoidance behavior in the context of information encountering was obtained, as shown in Figure 1. It should be noted that not all incidents contain every element in the process of avoidance behavior.

## 5. Discussion and Implication

### 5.1. Implications of the Phase in the Process

This study puts the avoidance behavior in the context of information encountering, which was complementary to previous information avoidance-related researches and contributed to the understanding of the information avoidance process.

#### 5.1.1. Effective Information Stimuli Help Reduce Information Avoidance

Avoidance behavior begins with the user’s attention to the information encountered. In terms of avoidance strategies, avoiding information sources was adopted most. This showed how attracting users’ attention during information dissemination, and thus facilitating their click behavior, was the first step in helping to realize the value of information. From the results of the analysis, the information that helped control and prevent diabetes could guide the user’s practice, could attract their attention, and trigger further examination. In the future, researchers can explore which topics, presentations, and information-organization methods are more conducive to attracting users’ attention and clicking, guiding better information flow and information value.

#### 5.1.2. Avoidance Behavior Can Be Intervened

Users’ avoidance behavior from avoidance to non-avoidance suggested that appropriate publicity and guidance would help users reduce avoidance of useful online diabetes information, facilitate the flow of online diabetes information, achieve the value of the information, and help change users’ unhealthy behaviors and improve their health. Users’ avoidance behavior from non-avoidance to avoidance meant that after successfully attracting users at the beginning, how to maintain user stability, reduce user churn, and enhance user stickiness should be considered.

In terms of avoidance intensity, many users showed a strong avoidance intention of topics such as diabetes complications, patients’ painful experiences, and scientific research, while users showed a general avoidance intention of most other topics. Since diabetes can lead to various complications, diabetic patients and family members of diabetic patients need to learn about diabetes complications, such as preventive measures for complications. During the dissemination of diabetes complications, more attention should be paid to the prevention of various complications. To attract users’ attention, a small amount of information about the serious consequences of complications can also be added. The intensity of user avoidance was dynamic, especially the change from general avoidance to strong avoidance, which indicated that when propagating diabetes-related information to users, it is necessary to ensure the quality of information and the credibility of the information source to reduce the loss of users.

### 5.2. Implications of the Influencing Factors

This study identified 14 factors that influenced the occurrence of avoidance behavior. They were further recognized as user-related, information-related, environment-related, and emotion-related factors. Targeted countermeasures can be conducted from user-related, information-related, and environment-related factors to reduce the occurrence of avoidance behavior.

#### 5.2.1. User-Related Factors

Demographic characteristics, including health status, personal/family disease history, and habits, were stable factors and difficult to change, and it was difficult to intervene in users’ avoidance behavior through them. On the other hand, health-behavior perception, perceived threat, perceived control, and information sufficiency was dynamic, and users’ avoidance behavior could be intervened through them.

(1) Health-behavior perception. When deciding whether to implement the content or suggestions of the online diabetes information encountered, general users, diabetic patients, and family members of diabetic patients would first consider barriers (i.e., perceived barriers) and benefits (i.e., perceived benefits). Online diabetes information will only be accepted and used by them if it is within an acceptable range of difficulty of implementation and utility.

(2) Perceived threat. Currently, the awareness rate of diabetes is still low. Many people thought that if there were no diabetic patients in their families, the possibility of suffering from diabetes was low, paying no attention to the prevention of diabetes in their daily life. It is necessary to increase the awareness of diabetes-related knowledge, enhance users’ awareness of the susceptibility and severity of diabetes, and help them form healthy living habits.

(3) Perceived control. Through interviews with patients and their families, we learned that elderly patients have a good mentality, pay attention to daily health care, and have good condition control. The newly diagnosed younger patients had large emotional fluctuations when facing diabetes (P15) and were prone to give rise to negative emotions when facing such information. Cases or stories can be used to explain to them that diabetes is manageable and to increase their confidence in coping with the disease.

(4) Information sufficiency. It was affected by information demand and knowledge reserve and was determined by the gap between the user’s knowledge reserve and information demand. When user information demand is greater than the current knowledge reserved, users tend to acquire knowledge. On the one hand, it is necessary to clarify which diabetes information users are interested in according to their usage habits. On the other hand, it is necessary to include new information in the recommendation process to increase their reading possibilities.

#### 5.2.2. Information-Related Factors

It includes information dissemination, information quality, and information overload.

(1) Information dissemination, including information source credibility, title description, information topic, and information presentation. From the results of the analysis, in addition to complications, severe or painful experiences caused by diabetes, and scientific research, other topics of online diabetes information were of high concern to the general users, diabetic patients, and family members of diabetic patients. In terms of complications, the information on how to prevent complications is more acceptable to users than the consequences caused by complications. In terms of scientific research, some users pay attention to the research that can be implemented. Diabetes-related severe or painful experiences might alert individuals to the seriousness of the disease and draw their attention. Therefore, it is necessary to consider a more acceptable way to present this information to users.

Title description. Users were more interested in viewing rigorous, professional, and focused titles. How titles are expressed can attract users’ attention and stimulate reading interest needs further verification. Existing research found that message framing and evidence types influenced users’ health information adoption and behavior change. In the future, we can explore whether message framing and evidence types can play a role in attracting users’ attention and reading online diabetes information, and compare whether there are differences between them.

Information sources’ credibility includes trusted information release channels and trusted information publishers. Most users preferred to read the content posted by experts, such as doctors and professors, on official platforms, such as hospital online platforms, WeChat official accounts, authentication Weibo, TikTok, etc., while a few users were more concerned with patients’ personal experiences and daily health care. Information distribution platforms can increase information filtering, ensure the authority of information publishers and published content, and enhance users’ trust in information sources, thus reducing direct avoidance caused by information sources and promoting information flow.

(2) Information quality. For different users, it is necessary to explore which information presentation method can achieve better communication effects. High-quality information with high readability, usefulness, and practicality will stimulate users’ interest in reading and increase the possibility of information being used; Low-quality information with poor readability, uselessness, and low practicability will directly lead to user avoidance. High information quality is the key to ensuring continuous information flow and access to information. Therefore, it is necessary to ensure information quality.

(3) Information overload, including high pushing frequency, large pushing quantity, and high similarity, are important factors that cause users to avoid it. The appearance of a large number of similar information will arouse users’ visual fatigue. To ensure better information dissemination, the platform should appropriately control the amount of information dissemination to reduce the cognitive burden of users.

### 5.3. Environment-Related Factors

In the browsing context, users are more likely to encounter online diabetes information and view it when browsing aimlessly. In the process of intelligent recommendation, a focus on the information push in the browsing context is recommended.

## 6. Conclusions and Future Research

This study focused on users’ avoidance behavior of online diabetes information under the background of information encountering. Diary and interview methods were used to collect data on the avoidance behavior of diabetic patients, family members of diabetic patients, and general users. Based on the thematic analysis method and grounded theory, the authors analyzed the process of users’ avoidance behavior and its influencing factors. The results show that the process of users’ avoidance behavior includes three phases: pre-encountering, encountering, and avoiding after encountering. First, browsing, searching, or social interaction provides the context for encountering. Second, the encountering occurrence consists of three steps–noticing the stimuli, reacting to stimuli, and examining the content. And third, to avoid the online diabetes information users encountered, users will adopt avoidance strategies, such as avoiding information sources, controlling attention, delaying access, forgetting information, and denying information, which is manifested as general avoidance and strong avoidance. It has positive, negative, or no effect on users. After avoiding, users will take actions such as returning to the pre-encountering activities, ending the current activities and further exploring and using the information encountered. Avoidance behavior and avoidance intensity are dynamic. Avoidance behavior is affected by user-related factors, information-related factors, environment-related factors, and emotion-related factors.

The limitation of this study is that it only reveals the process and influencing factors of avoidance behavior of online diabetes information from a qualitative perspective and does not further verify the mechanism of the process and influencing factors of avoidance behavior. The data obtained from the interview comes from the user’s memory, which is less complete than the diary, leading to an incomplete process of avoidance behavior based on interview data. In the future, we need to conduct in-depth research on the avoidance behavior of health information from the perspective of research content and research methods and conduct an in-depth analysis of different phases of avoidance behavior from the aspect of the content. For example, in the pre-encountering phase, we can explore the contributing factors of health information encountering. In the encountering phase, we can analyze how users’ cognition evolves and what characteristics of health information will lead to users’ approach or avoidance. In the avoiding after encountering phase, we can explore how factors act on users’ avoidance behavior and the mechanism of emotional factors on users’ avoidance behavior, and weigh the influence of individual beliefs on the selection and interpretation of health information. In terms of research methods, qualitative data can be collected by combining observation methods, and objective data on users’ avoidance behavior can be obtained by eye-tracking experiments. In addition, data analysis can be combined with qualitative comparative analysis and simulation analysis to systematically reveal the complex relationship between the influencing factors of avoidance behavior and enhance the reliability of the conclusion.

## Figures and Tables

**Table 1:** Code example of the avoidance behavior process.

Original Descriptions	Notes	Initial Code	Formal Code	Incident
Browsing QQ Zone to see the status of students	Browsing QQ Zone	QQ Zone	QQ	I3
Browsing the Weibo homepage	Browsing Weibo	Weibo	Weibo	I7
Browsing an article on WeChat clove doctor	Browsing WeChat public account	WeChat public account	WeChat	I9

**Table 2:** Analysis results of sub-themes and main themes of the avoidance behavior process.

Sub-Themes	Main Themes	Phase
Social media	Online environment	Pre-encountering
Search engines		
Video platforms		
Other online environments		
Pre-encountering positive emotions state	Pre-encountering emotional state	
Pre-encountering negative emotions state		
Browsing	Foreground activities	
Searching		
Social interaction		
Study	Activity scenario	
Work		
Everyday life		
Health irrelevant information	Pre-encountering information topic	
General health information		
Topic of the information encountered	Stimulus noticed	Encountering
Information presentation		
Immediately avoid	Reaction to stimulus	
Further access		
Content description of the information encountered	Content examined	
Information content presentation		
Not satisfied	Reaction after examining the information	
Unable to implement		
Not applicable		
Difficult to implement		
Cognitive conflict		
Negative emotion after examination	Emotional state after examination	
Positive emotion after examination		
Avoiding information source	Avoiding strategy	Avoiding after encountering
Controlling attention		
Delaying access		
Forgetting information		
Denying information		
General avoidance	Avoiding intensity	
Strong avoidance		
Positive effect	Effect of avoidance behavior	
Negative effect		
No effect		
Positive emotion after avoiding	Emotional state after avoiding	
Negative emotion after avoiding		
Return to the initial activity	Post-avoidance behavior	
End all activities		
Using the information encountered		
Further exploring		

**Table 3:** Code example of factors influencing the occurrence of the avoidance behavior.

Original Sentences	Notes	Initial Concept	Category	Participant
At present, it didn’t work around me	Practical for me	Practical	Practicability	P3
I didn’t feel very interested in other contents, and I didn’t understand them very well	Didn’t interest in and understand	Too academic	Readability	P4
This tweet is very helpful for guiding patients to take medicine	Helpful	Useful	Usefulness	P5

**Table 4:** Coding results of core categories and main categories.

Categories	Main Categories	Core Categories
G1 Practicality	M1 Information quality	N1 Information-related factors
G2 Usefulness		
G3 Readability		
G4 Novelty		
G5 Too many information feeds	M2 Information overload	
G6 High similarity of information		
G7 Source reliability	M3 Information dissemination	
G8 Information title description		
G9 Information topic		
G10 Information presentation		
G11 Health status	M4 Demographic characteristics	N2 User-related factors
G12 Personal/family disease history		
G13 Habit		
G14 Perceived difficulty	M5 Health-behavior perception	
G15 Perceived utility		
G16 Severity	M6 Perceived threat	
G17 Susceptibility		
G18 Well controlled of disease	M7 Perceived control	
G19 Information needs	M8 Information sufficiency	
G20 Insufficient knowledge reserve		
G21 Browsing	M9 Context type	N3 Environment-related factors
G22 Searching		
G23 Social interaction		
G24 Study		
G25 Work		
G26 Everyday life		
G27 Public places	M10 Behavior place	
G28 Busy	M11 Time pressure	
G29 Fragmented time		
G30 Subjective norms	M12 Social factors	
G31 Pre-encountering negative emotional state	M13 Pre-encountering emotional state	N4 Emotion-related factors
G32 Post-encountering negative emotional state	M14 Post-encountering emotional state	
G33 Avoiding information sources	M15 Avoidance behavior	N5 Avoidance behavior
G34 Controlling attention		
G35 Delaying access		
G36 Forgetting information		
G37 Denying information		

## Data Availability

The data of the work can be provided by the corresponding author upon request.
